# Curing the pandemic of misinformation on COVID-19 mRNA vaccines through real evidence-based medicine - Part 2

**DOI:** 10.4102/jir.v5i1.72

**Published:** 2022-09-26

**Authors:** Aseem Malhotra

**Affiliations:** 1 Public Health Collaboration, London, United Kingdom

**Keywords:** COVID-19, mRNA vaccine, cardiac arrests, real evidence-based medicine, shared decision making

## Abstract

**Background:**

Authorities and sections of the medical profession have supported unethical, coercive, and misinformed policies such as vaccine mandates and vaccine passports, undermining the principles of ethical evidence-based medical practice and informed consent. These regrettable actions are a symptom of the ‘medical information mess’: The tip of a mortality iceberg where prescribed medications are estimated to be the third most common cause of death globally after heart disease and cancer.

**Aim:**

To identify the major root causes of these public health failures.

**Methods:**

A narrative review of both current and historical driving factors that underpin the pandemic of medical misinformation.

**Results:**

Underlying causes for this failure include regulatory capture – guardians that are supposed to protect the public are in fact funded by the corporations that stand to gain from the sale of those medications. A failure of public health messaging has also resulted in wanton waste of resources and a missed opportunity to help individuals lead healthier lives with relatively simple – and low cost – lifestyle changes.

**Conclusion:**

There is a strong scientific, ethical and moral case to be made that the current COVID vaccine administration must stop until all the raw data has been subjected to fully independent scrutiny. Looking to the future the medical and public health professions must recognise these failings and eschew the tainted dollar of the medical-industrial complex. It will take a lot of time and effort to rebuild trust in these institutions, but the health – of both humanity and the medical profession – depends on it.

**Contribution:**

This article highlights the importance of addressing metabolic health to reduce chronic disease and that insulin resistance is also a major risk factor for poor outcomes from COVID-19.

## A pandemic of misinformation

What has become clear with regard to the coronavirus disease 2019 (COVID-19) vaccines is that we have a pandemic of misinformed doctors and a misinformed and unwittingly harmed public. Coercively mandating these COVID-19 vaccinations (most certainly not an evidence-based policy) has been a particularly egregious mis-step, especially in the light of clear indicators suggesting that the use of these pharmaceutical interventions – especially in younger age groups – should have been suspended. Such policies continue to undermine the principles of ethical evidence-based medical practice and informed consent, to the detriment of optimising patient outcomes.

In his 2017 paper, ‘How to survive the medical misinformation mess’, Professor John Ioannidis and colleagues highlight that:

[*M*]ost clinical trial results may be misleading or not useful for patients. Most guidelines (which many clinicians rely on to guide treatment decisions) do not fully acknowledge the poor quality of data on which they are based. Most medical stories in mass media do not meet criteria for accuracy, and many stories exaggerate benefit and minimise the harms.^1^ (p. 1)

A senior doctor in regular contact with the United Kingdom’s (UKs) Chief Medical Officer Professor Chris Whitty recently expressed concerns to me that he felt most of his colleagues in leadership positions influencing health policy may not be critically appraising the evidence and instead are relying on media stories on COVID-19 and the vaccine. This is consistent with the admission of Rochelle Walensky, the former chair of the Centers of Disease Control (CDC), whose optimism in the efficacy of Pfizer’s COVID-19 vaccine came from reading a CNN news story, which was an almost verbatim reproduction of Pfizer’s own press release.^2^

Has the UKs Chief Medical Officer Professor Chris Whitty critically appraised the evidence? Recently, he publicly shared a letter^3^ outlining the importance of healthcare staff to become vaccinated against COVID-19, which was neither comprehensive nor consistent with the totality of the evidence: ‘The COVID-19 vaccines are safe and effective’. It would have been more accurate to state that ‘the vaccine is not completely safe and not anywhere close to being as effective as we’d hoped for. Not even in the same ball park when compared to the efficacy and safety of traditional vaccines’.

Professor Chirs Witty stated:

Our professional responsibility is to get the covid vaccines as recommended to protect our patients’.^3^

He should have said as far as omicron is concerned, ‘the vaccine offers little to no protection against infection. Data on the delta variant also revealed that once infected there is no significant difference in transmission rates between the vaccinated and unvaccinated individuals.

Professor Whitty’s statements are especially surprising given that the CEO of Pfizer has stated that in realtion to omicron ‘We know that the two doses of a vaccine offer very limited pretection, if any’.^5^

Could it be that Professor Whitty is also a victim of the medical misinformation mess?

There are four key drivers and seven sins that are at the root of the medical misinformation mess:

- Drivers:
  ▪ Much published medical research is not reliable or is of uncertain reliability, offers no benefit to patients or is not useful for decision makers;
  ▪ Most healthcare professionals are not aware of this problem;
  ▪ Even if they are aware of this problem, most healthcare professionals lack the skills necessary to evaluate the reliability and usefulness of medical evidence; and
  ▪ Patients and families frequently lack relevant, accurate medical evidence and skilled guidance at the time of medical decision making.^1^
- Sins:
  ▪ Biased funding of research (that’s research that’s funded because it’s likely to be profitable, not beneficial for patients)
  ▪ Biased reporting in medical journals
  ▪ Biased reporting in the media
  ▪ Biased patient pamphlets
  ▪ Commercial conflicts of interest
  ▪ Defensive medicine
  ▪ An inability of doctors to understand and communicate health statistics.^6^

Ioannidis and colleagues highlight that:

‘Ignorance of this problem, even at the highest levels of academic and clinical leadership, is profound’^1^

Compounded over several decades, these upstream and downstream risk factors for misinformation have had a devastating effect in the healthcare environment we find ourselves in today. Over-prescription of drugs is considered such a public health threat that two leading medical journals in the past 10 years (the *BMJ* and *JAMA Internal Medicine*) have launched campaigns to reduce the harms of too much medical intervention. According to the cofounder of the Cochrane Collaboration, Peter Gøtzsche, prescribed medications are the third most common cause of death globally after heart disease and cancer.^7^ This is not surprising when one understands that most published research is misleading specifically where benefits from drug trials are exaggerated, and harms downplayed (Box 1^8^).

BOX 1 Major limitations in the interpretation, external validity and usefulness of drug industry-sponsored clinical trials.

1. Trials are conducted of a study drug against a treatment known to be inferior
2. Use multiple endpoints in the trial and select for publication those that give favourable results
3. Do multicentre trials and select for publication results from centres that are favourable
4. Conduct subgroup analyses and select for publication those that are favourable
5. Present results that exaggerate the benefit – for example, use of relative risks as opposed to absolute risks
6. Conduct trials on subjects that are unrepresentative of the patient population
7. Conflate primary and secondary endpoints in the published report
8. Conceal unblinded patients and include them in efficacy analyses for publication
9. Exclude placebo responders in the wash-out phase of the trial
10. Delay publication of negative trial results until positive trial results are published
11. Conceal negative trial results whilst publishing only positive trial results
12. Conceal serious adverse events
13. Fail to distinguish clinical from statistical significance

*Source*: Adapted from Jureidini J, McHenry L. The illusion of evidence based medicine. Adelaide: Wakefield Press; 2020

If a doctor is making clinical decisions on biased information, it will lead (at best) to suboptimal outcomes and (more concerningly) harm to patients.

### Shortcomings of the medical profession

According to Professor Carl Heneghan and urgent care General Practitioner, the director of the University of Oxford’s Centre of Evidence-Based Medicine: ‘with every intervention you do as a doctor you must ask yourself two questions: how much difference does it make? How do I know this?’^9^

Building on the Academy of Medical Royal Colleges Choosing Wisely campaign,^10^it is instructive to note that the General Medical Council in 2020 issued guidance on the duty of doctors to engage in Shared Decision Making with patients, underpinned by informed consent.^11^

There are six components essential to informed decision making: (1) description of the nature of the decision; (2) discussion of alternatives; (3) discussion of risks and benefits (in absolute terms); (4) discussion of related uncertainties; (5) assessment of the patient’s understanding; and (6) elicitation of the patient’s preference.

If the administration of the vaccine did not adhere to these principles (which is likely widespread, consistent with historical evidence^12^), then it is also a significant breach of General Medical Council duties of a doctor to ‘give patients the information they want or need in a way that they can understand’.^13^

It is instructive to note that the greater the financial interests in a given field, the less likely the research findings are to be true.^14^ As has been already demonstrated in Part 1^15^ of this article, mandating a novel emergency use authorisation vaccine to non-vulnerable people has little to no effect on preventing infection and serious illness, therefore does not have any scientific validity, and therefore breaches the principles of informed consent. It does, however, dramatically enhance the profits of the manufacturer. By expanding the uptake of the mRNA vaccine to the majority of the population that are very low risk of serious complications from COVID-19 but are more likely to suffer serious and/or life-threatening adverse events such as myocarditis or sudden cardiac death, Pfizer has generated tens of billion dollars in revenues to date, making it one it one of the most lucrative products in history. If policymakers had focussed more on protecting the vulnerable – and doctors had been given the opportunity to practice shared decision making with patients using transparent communication of risk and benefit – patient outcomes would likely have been significantly improved,^16^ but the drug companies’ profits would likely have been a tiny fraction of what they actually generated. As former Editor of the *New England Journal of Medicine* Dr Marcia Angell has previously pointed out ‘the real battle in healthcare is one of truth versus money’.^17^

### Institutional corruption and erosion of public trust

Institutional corruption is defined as an institution’s deviation from a baseline of integrity.^18^ There is a long-documented history (both through studies and lawsuits) of the strategies in which drug companies hide, ignore or misrepresent evidence about new drugs. Distortion of medical literature and misrepresentation of data by companies keen to expand the marketplace for their product may result in overprescribing with predictable consequences of millions of patients suffering from avoidable adverse reactions.

Prior to 2020 there already existed gross shortcomings in the medical–industrial complex – there has been too much pharmaceutical industry influence on clinical decision making. This has not gone unnoticed, resulting in a growing crisis of trust in medical research: a report by the Academy of Medical Sciences in 2017 revealed that 82% of GPs and 63% of the public did not believe the results of pharmaceutical industry-sponsored research to be unbiased.^19^ Similarly, only 37% of the public trust medical research compared to 65% who trust the experience of their friends and family.^20^

This growing lack of trust – most recently exacerbated by coercion, vaccine passports and little mainstream media coverage of an unprecedented scale of reported vaccine harms in the population – has been most recently exemplified by 8 million people in the UK refusing to take the COVID-19 booster shot. In addition, with all the attention on COVID-19 (which poses almost zero risk to children in its current omicron form), diverts attention away from, and even worse raises the suspicion of, more efficacious and safe interventions such as the measles, mumps, rubella (MMR) vaccine. Indeed, in the UK MMR vaccination rates have hit their lowest for 10 years.

### Failure of regulation and research misconduct

Authorities want the public to ‘trust the science’, but vaccine manufacturers have successfully negotiated deals with several major governments globally that indemnify them against any financial liability in the event of vaccine-related harm. Interestingly, India, the world’s largest democracy, refused to grant Pfizer indemnity from harms for its vaccine. An Indian government source told Reuters that:

[*T*]he whole problem with Pfizer is the indemnity bond. Why should we sign it? If something happens, a patient dies, we will not be able to question them [*Pfizer*]. If somebody challenges in a court of law, the central government will be responsible for everything, not the company.^21^ (p. 1)

Pfizer walked away from the Indian market rather than undertake a local safety and immunogenicity study.^22^

It is important to first understand that drug companies have a fiduciary obligation to deliver profits to their shareholders, not any legal responsibility to provide you with the best treatment. At a talk at the Centre of Evidence-Based Medicine in Oxford in 2014, Peter Wilmshurst said the real scandal is that many of those with a responsibility to patients and scientific integrity (doctors, academic institutions and medical journals) often collude with industry for financial gain.^23^ It is this very industry that has been found guilty of the most egregious corporate crimes: between 2003 and 2016 the top 11 pharmaceutical companies paid $28.8 billion in fines just within the United States (US),^24^ much of it for criminal activity such as the illegal marketing of drugs, manipulation of results and hiding data on harms. As pointed out in the *BMJ*, since then no systemic changes have been made to mitigate these harms.^9^

In an international survey of respondents from higher education institutions, 14% admitted to knowing a colleague who fabricated, falsified and modified data, and 34% of scientists report questionable research practices that included selective reporting of clinical outcomes in published research and concealing conflicts of interest.^25^ An egregious documented case of research misconduct involved a prominent Dutch physician whose work influenced the European Society of Cardiology guidelines on the use of beta blocker drugs in non-cardiac surgery. He was dismissed from Erasmus University for ‘violations in academic integrity’, including using ‘fictitious data’ in research. It’s estimated that these guidelines increased patient mortality by 27% resulting in 800 000 excess deaths across Europe over an 8-year period.^26^

In evidence submitted to the UK parliamentary science and technology review into research integrity committee in 2017 (Chaired by Sir Norman Lamb), Dr Peter Wilmshurst lists a number of risk factors that drive research misconduct in British institutions (see Box 2^27^). His solution, which I agree with, would be to ensure that serious forms of research misconduct are made into criminal offences with meaningful sanctions and that allegations of such activity should be investigated by an independent body with legal powers.^27^

BOX 2 Written evidence from Dr Peter Wilmshurst to UK Parliamentary Science and Technology Research Integrity Committee (June 2018).

Academic institutions bear responsibility for the pressure to publish for career advancement that can result in research misconduct.

A record of prominent publication is likely to attract future funding, which institutions demand, and good publicity, which institutions desire.

Other pressures for misconduct come from the association of academic institution with industry, such as when investigators or their institutions hold patents or shares, or they receive payments from industry, so that there is financial pressure to publish research that will be profitable for the company and to suppress ‘negative’ findings.

Some publications are simply organised criminal activities, which may be at the behest of sponsors, when prominent academics are paid large sums of money to publish false data by industry, or a sponsor may be one of the victims, when payments for conducting research are made to ‘investigators’, who simply fabricate data.

Medical journals have financial pressures to publish positive findings of research on drugs and medical devices, because their manufacturers buy reprints of the papers for distribution to doctors and they pay for advertisements linked to articles favourable to their product.

Academic institutions and journals depend on the public belief in the integrity of science, so they are unwilling to admit the seriousness and frequency of research misconduct.

To protect their reputations academic institutions conceal research misconduct, destroy evidence and silence whistle-blowers.

Journals are reluctant to admit that they published flawed research, so they commonly refuse to publish failures to replicate.

Fear of a libel action contributes to the failure to expose research misconduct.

Investigation of research misconduct may be difficult because there may be international collaboration between investigators, many of whom do not see the full data, and the resulting publications may be in journals that are published in countries where none of the investigators work.

The bodies that investigate research misconduct in the UK (such as the GMC and UKRIO) are hampered by a desire to play down the problem, by lack of proper forensic skills when investigating, by inconsistent interpretation of rules and by inadequate powers to compel the cooperation of academic institutions and journals.

Because lenient sanctions are imposed, institutions believe that the misconduct is not very serious, and potential research fraudsters are not deterred.

*Source*: Wilmshurst P. Written evidence [homepage on the Internet]. 2017 [cited 2022 Jun 5]. Available from: http://data.parliament.uk/writtenevidence/committeeevidence.svc/evidencedocument/science-and-technology-committee/research-integrity/written/68813.html

GMC, General Medical Council; UKRIO, United Kingdom Research Integrity Office.

One researcher at a prestigious UK institution contacted me to inform me that in his cardiology department a group of academics were deliberately suppressing research that revealed that the mRNA vaccine was shown to significantly increase coronary risk as determined by cardiac imaging as compared to the unvaccinated. The chair of the group expressed concerns that publishing the data may result in loss of funding from the pharmaceutical industry.^28^ After I had alluded to this on GB News, the whistle-blower informed me that non-disclosure agreement letters were sent to all members of the team involved in this particular area of research.

## Evidence-based medicine and COVID-19 vaccine roll-out

Neither the drug regulators nor the vaccine manufacturers have yet to share all the raw data from the pivotal trials for the COVID-19 vaccines.^29^ The raw data from clinical trials comprise thousands of pages that have yet to be released for independent scrutiny. This is important because historically when independent researchers have on occasion gained access to this data then it can completely overturn the conclusions of the published trials: A case in point is Tamiflu.^30^ Getting access to clinical case reports for Tamiflu ultimately revealed that the drug was no more effective than paracetamol for influenza and also came with small but significant harms. The UK government had spent half a billion dollars stockpiling a drug that in effect proved to be useless despite claims by the manufacturers (Roche, Basil, Switzerland) that it shortened the duration and severity of the illness. The independent researchers who were able to analyse the data concluded that all industry-sponsored research should be considered marketing until proven otherwise.

It is against this backdrop that transparency advocates sued the Food and Drug Administration (FDA) to gain access to the data upon which the Pfizer (BNT162b2) vaccine was granted emergency use authorisation.^31^ The FDA wanted a US Federal court judge to allow the agency 55 years to release this data.^32^ Why would the FDA – ‘which is responsible for the oversight of more than $2.7 trillion in consumption of food, medical products, and tobacco’^33^ – do this? Secrecy should never surround any public health intervention. The lawyer acting on behalf of the plaintiff Aaron Siri reported that:

[*T*]he government also sought to delay full release of the data it relied upon to license this product until almost every American alive today is dead. That form of governance is destructive to liberty and antithetical to the openness required in a democratic society.^31^

Instead, the judge ordered the FDA to release the data over a period of eight months after all commercially sensitive information has been redacted.

A major risk factor for failure to protect the public from such harms is lack of independence of the regulator. The FDA’s Centre for Drug Evaluation Research (CDER) receives 65% of its funding from the pharmaceutical industry (mainly in the form of user fees).^34^ For example, as part of the approval process for its COVID-19 vaccine, Pfizer made a wire transfer to the FDA of $2 875 842 million in May 2021^35^ under the *Prescription Drug User Fee Act* of 1992.^36^ Full FDA approval for Pfizer’s COVID-19 injection duly followed in August 2021^37^ despite recent evidence emerging that the original RCT data suggested a greater risk of serious adverse events from the vaccine than from hospitalisation because of COVID-19.

Separate analyses have revealed the overwhelming majority of new drugs that have been approved by the FDA in the past few decades have later been shown to be just copies of old ones, which is not surprising when one understands that drug companies spend 19 times more on marketing than they do on researching new molecular entities, which all contributes to considerable waste. Between 2000 and 2008 of the 667 drugs approved by the FDA, only 11% were found to be truly innovative. In the US it’s estimated that 30% – 50% of healthcare activity brings no benefit to patients. Extraordinarily, a survey of FDA scientists revealed 70% of them did not feel the FDA had the resources to perform effectively in its mission in ‘protecting public health … and helping the public get accurate science-based information to use medicines and foods to improve their health’.^38^

An analysis of every new drug product approved in France between 2002 and 2011 revealed only 8% offered some advantages and double that amount – at 15.6% – were found to be more harmful than beneficial with the majority of other new drugs being essentially copies of old ones contributing to a colossal waste of public money.^18^ Similar conclusions have been drawn in Canada and Holland. In my opinion the evidence is overwhelming that the overall net effect of the pharmaceutical industry in the last few decades on society and population health has been a hugely negative one.

## COVID-19 vaccination in lower risk individuals

Irrespective of the merits of inoculating higher risk groups where a small but significant benefit may exist against the original Wuhan strain, vaccinating lower risk children in the name of preventing asymptomatic transmission has no strong scientific validity and therefore exposes them to possible harm.

In the UK the Office for National Statistics has revealed an as yet unexplained significant increase in deaths over the 5-year average in 15- to 19-year-old children since May 2021. Given what we now know of potential harms especially in relation to myocarditis, myocardial infarction and sudden cardiac death (even in 16- to 39-year-olds) has the COVID-19 vaccine been excluded as a possible cause?^39^

In September 2021, the Joint Committee on Vaccination and Immunisation (JCVI) made a controversial recommendation that the Pfizer/BioNTech vaccine is marginally beneficial for 12- to 15-year-old children.^40^ The Medicines and Healthcare products Regulatory Agency (MHRA, the UK’s equivalent of the FDA) had previously stated that:

[*T*]hey have carefully reviewed clinical trial data for Pfizer/BioNtech vaccine in over 2000 children aged 12–15 years of age and have concluded that the benefits of this vaccine outweigh any risk and that it is effective and acceptably safe in this age group … No new side effects were identified and the safety data in children was comparable to that seen in young adults. As in the young adult age group, the majority of adverse events were mild to moderate, relating to reactogenicity (e.g. sore arm and tiredness).^41^ (p. 1)

Is this in keeping with the totality of the evidence?

Award winning investigative science journalist Maryanne Demasi published the harrowing story of one of those trial participants, 12-year-old Maddie De Garay. After experiencing severe abdominal pain followed by seizures she was admitted to hospital and is now left permanently disabled, wheelchair bound and fed through a nasogastric tube. In Pfizer’s trial they reported her adverse effect as mild: stomach upset.^42^

It is important to emphasise that the risk of death from COVID-19 in a 12- to 15-year-old is close to zero at 1 in 76 000. In keeping with the principles of ethical evidence-based medical practice through shared decision making, parents need to be told that there is no high-quality data in children that the vaccine will prevent infection, transmission, serious illness or death but may come with serious side effects of myocarditis – particularly in young males where it occurs in up to 1 in 2700^43^ – and serious disability as a general principle of transparent communication of risk and informed consent: without understanding the numbers involved the public is vulnerable to their hopes and anxieties being exploited by political and commercial interests.

### Could financial interests be biasing the recommendations?

On its website the MHRA declares that the majority of its funding comes from the pharmaceutical industry and £3 million (UK pounds) from the Bill and Melinda Gates Foundation (BMGF). Are policymakers and the public aware that the foundation’s corporate stock endowment is heavily invested in food (including McDonald’s and Coca-Cola) and pharmaceutical companies, directly and indirectly? As pointed out in a 2009 *Lancet* paper, the funders’ priorities are often driven by personal interests, not the health priority interests of the recipient country.^44^ ‘The BMGF’s portfolio of pharmaceutical companies calls for attention given Mr Gates’ personal belief in the role of patents as motors for innovation in medicines and medical technology’.^45^

Obesity researcher Dr Zoe Harcombe has also investigated the financial ties that could potentially be biasing the view of the joint committee for vaccines and immunisation and discovered that the subcommittee members work for organisations that receive in total $1bn from the BMGF.^46^ It is also worth noting that Professor Wei Shen Lim, chairman of the JCVI vaccine subcommittee, has direct responsibility for material levels of funding received by his department from Pfizer.^47^ This is not in any way suggesting that the JCVI have acted in an improper way, but when confidence in an organisation such as the JCVI is imperative it’s essential that there should be no perceptions of conflicts of interest. The systems of selection of panellists, the scrutiny of evidence and the methodology and openness of their recommendations need to be beyond reproach.

### The most proximate cause of detrimental health outcomes: Corporate power and the commercial determinants of health

**FIGURE 1:**
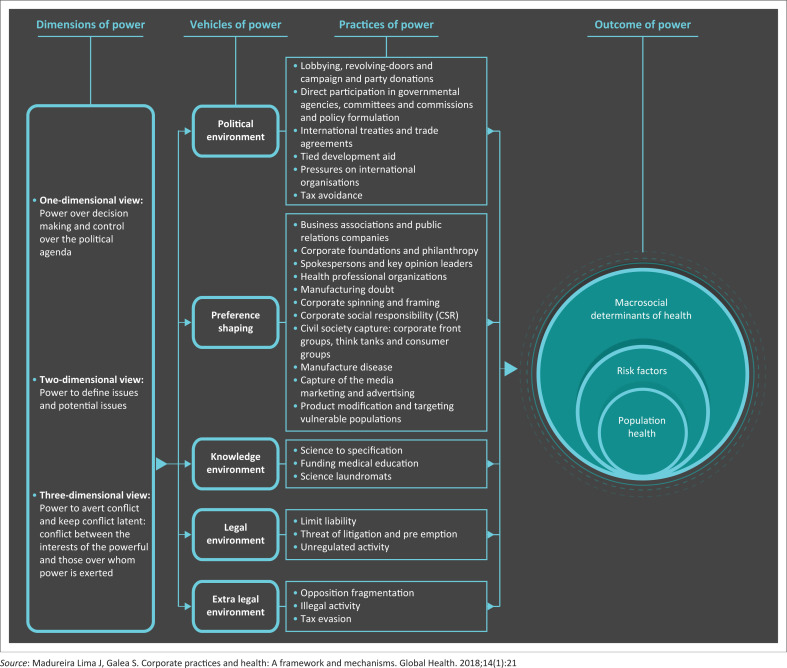
Diagram of dimensions, vehicles, practices and outcomes of power.

The commercial determinants of health are best defined by ‘strategies and approaches adopted by the private sector to promote products and choices that are detrimental to health’.^48^ Corporations exert their power by a combination of factors including intellectual exploitation. This includes the ability to define the dominant narrative: set the rules and procedures by which society is governed; determine the rights, living and working conditions of ordinary people; and take ownership of knowledge and ideas^49^ (see Figure 1^45^). It appears that in the case of the mRNA vaccine, Pfizer has at least to some degree taken advantage of this corporate framework strategy by shaping the knowledge environment (Pfizer was responsible for the design and conduct of the trial, data collection, data analysis, data interpretation and the writing of the manuscript), the political environment (lobbying), preference shaping (corporate foundations and philanthropy, spokespersons and key opinion leaders, capture of the media), the legal environment (limit liability) and the extra-legal environment (opposition fragmentation by de-platforming critics of the current dominant narrative that the vaccine is safe and effective).^45^ Consequently, it has made tens of billions of dollars in revenue from a product that in comparison with time-tested traditional vaccines and most other drugs has extremely poor efficacy and unprecedented reports of serious harms.

### Biased reporting in the media and censorship of legitimate scientific debate

Corporations are able to shape preferences and frame the dominant narratives on the determinants of health, through unchecked invisible power. One pathway is through the ownership of mass media. The global media market is dominated by seven corporations and chains that own 80% of the newspapers in the US.^50^ The grants paid to global media companies by the BMGF are notable – for example, The Guardian Media Group has been in receipt of over $12m in grants from the BMGF over the last 12 years. Control over advertising in print and broadcast media also has an influence over editorial decisions. Most health journalists (including a number I have spoken to) are generally unaware that the information they obtain for stories has been deliberately shaped by the private interests of manufacturers and ‘research’ universities.

The BBC, though seemingly not directly influenced by industry interests, has traditionally been seen by some as the UK’s most trusted media source. Its coverage of issues surrounding COVID-19 has in my view (possibly through additional government pressure) been extremely poor and – specifically on issues surrounding the vaccine – grossly negligent. During a recent report on tennis player Novak Djokovic explaining his decision to not take the vaccine until he has more information on its benefits and harms, a reporter asked the question ‘how much more information does he need?’. The reporter failed to mention the fact that Djokovic has had COVID-19 and that evidence suggests that natural immunity offers *significant* protection against reinfection and severe disease, and that systemic side effects are almost threefold more likely in those with natural immunity who subsequently get vaccinated. Furthermore, the BBC falsely framed a guest on popular podcast host Joe Rogan, Dr Robert Malone, as a ‘known anti-vaxxer, who is against vaccinating kids’, failing to mention that Dr Malone is a co-inventor of the very technology that led to the vaccine, has spent 20 years in vaccine development at US government level and was one the first to actually receive two shots of the Moderna jab. The BBC also strangely failed to cover perhaps one of the most significant stories of the pandemic published in one of the most respected and influential medical journals in the world: An investigation by the *BMJ* revealed evidence of poor practices at a contract research company involved in Pfizer’s pivotal COVID-19 vaccine trial. A regional director employed at one of the trial sites in Texas, US, documented evidence that Pfizer falsified data, unblinded patients, employed inadequately controlled vaccinators and was slow to follow up on adverse events. The very same day that she emailed her complaint to the FDA she was fired from her position.^51^ She subsequently commenced litigation under whistle-blower legislation for fraud against Pfizer on behalf of the American Government (and the people of the US). Pfizer’s motion to dismiss the case (which apparently did not sway the judge) was based on the fact that the FDA had not acted on her (or any other) complaints, hence the allegations were not material to the Government.

In the US, Senator Ron Johnson, who conducted hearings with healthcare professionals who were presenting data on clear, substantial and very common adverse effects from the mRNA jabs, which deserved widespread public attention, said ‘the mainstream media are co-conspirators in this political dirty trick. Will they be held accountable for their role in this deception’?^52^

Social media platforms continue to be guilty of spreading misinformation. Their business model that focusses on increasing engagement at any cost makes society increasingly lose access to the truth and worsens our capacity for empathy as individuals, sowing even greater division and hostility. The so-called ‘fact checkers’ have censored anything that challenges the prevailing mainstream narrative (the establishment is trustworthy, and the vaccines are completely safe). They even labelled the *BMJ*’s investigation into potential fraud in Pfizer’s pivotal trial as misinformation and stopped users sharing the story on their platform. A letter from the journal’s current and former editor in chief to Mark Zuckerberg calls into question the integrity of Facebook’s fact checkers:

[*R*]ather than investing a proportion of Meta’s substantial profits to help ensure the accuracy of medical information shared through social media, you apparently delegated responsibility to people incompetent in carrying out this crucial task.^53^ (p. 1)

It has also come to light that Facebook has partnered with drug company Merck in deciding what content should be censored on its platform in relation to COVID-19 and the vaccine.^54^ Is Facebook aware that Merck paid one of the largest fines in US history for being found guilty of fraud in relation to their pain killer Vioxx?^55^ Not only did an investigation reveal that the drug did not reduce gastric bleeds (their original key selling point) in comparison with ibuprofen, but it significantly increased the risk of heart attacks and strokes, estimated to have caused excess deaths of between 40 000 and 60 000 Americans over a 5-year period.^56^

### Improving metabolic health

Failure of public health messaging and policies to help individuals to improve their lifestyles during the pandemic represents a missed opportunity to mitigate harms from respiratory diseases such as COVID-19. After age, the biggest risk factor for worse COVID-19 outcomes has been obesity and conditions related to excess body fat. More than 90% of the deaths from COVID-19 occurred in countries where more than 50% of the population is overweight or obese. The United Kingdom’s biobank data during the first wave revealed a more than fourfold higher risk in hospitalisation from COVID-19 depending on lifestyle factors. For example, a non-smoking adult in their mid-fifties with a normal body mass index (BMI) and obtaining adequate physical activity levels had a 1 in 1521 chance of being admitted to hospital after contracting COVID-19, whereas an obese, smoking, sedentary person’s risk was 1 in 327.^57^

Postulated pathophysiological mechanisms of risk and complications from infection include an array of markers that have insulin resistance and chronic inflammation at the root.

**FIGURE 2:**
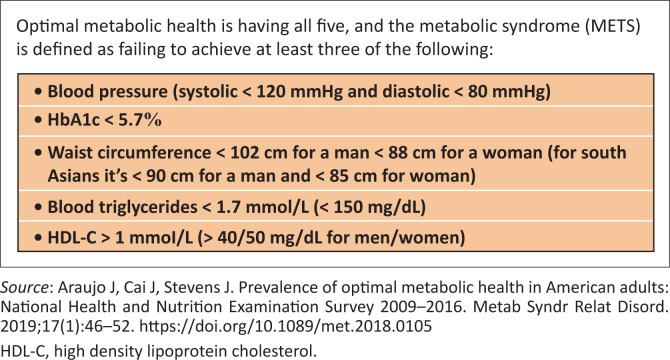
Markers of metabolic health.

Even a single high blood glucose reading in non-diabetics (a marker of insulin resistance) admitted to hospital has been shown to be associated with worse outcomes.^58^ It has also recently emerged in the UK that of the 175 256 deaths associated with COVID-19 (2020–2021 inclusive) less than 10% (17 371) had COVID-19 as the only cause on the death certificate suggesting that the risk to those individuals with optimal metabolic health from COVID-19 (Figure 2^59^) was significantly smaller, as per the results of the aforementioned UK biobank study.^60^

The government and medical authorities should have made it a priority to emphasise the importance of eliminating ultra-processed foods and low-quality carbohydrates to reduce risk. They could have made the public aware that reversal of metabolic syndrome has been shown to occur in up to 50% of patients – independent of weight loss – within four weeks of dietary changes alone.^61^

The coronavirus disease 2019 was a momentary crisis that exploited a slow pandemic of poor metabolic health (see Figure 2^59^), which is also the predominant root cause behind the major chronic diseases that have been putting healthcare systems around the world under increasing strain for decades. It is estimated that healthier lifestyles would (in absolute terms) *potentially* eliminate 40% of cancers and 75% of cardiovascular disease and type 2 diabetes.^63^

Optimising metabolic health would not just improve immune resilience but also reduce the burden of heart disease, type 2 diabetes, cancer and dementia. Learning lessons from tobacco control, policy changes that target the availability, acceptability and affordability of ultra-processed food and drink and low-quality carbohydrates would significantly reduce the burden of obesity, related metabolic diseases and likely optimise immune resilience in populations within a few years (see Box 3^62^).

BOX 3 Policies to curb obesity and lifestyle-related disease.

1. Taxation of all ultra-processed foods and drinks needs to be enforced with the money gained going directly to subsidise whole and minimally processed foods such as fruit and vegetables
2. All medical students and doctors need to have adequate training in nutrition and lifestyle medicine
3. Every doctor should be measuring the metabolic health of their patients and making lifestyle prescriptions specifically linked to diet, physical activity and stress reduction to improve those health markers as their first-line intervention before the use of medication
4. Compulsory nutrition education and cooking skills introduced into all school curriculums
5. All hospital chief executives need to be made accountable for allowing the sale of ultra-processed food on hospital grounds, as it continues to harm the health of staff and patients and legitimises the acceptability of such food consumption to the wider public
6. A ban on advertising of all ultra-processed food and drink on television and online demand services
7. A public education campaign is needed to help consumers understand what ultra-processed food is and the harm it causes
8. A complete ban and dissociation of ultra-processed food and drink sponsorship of sports teams and sporting events
9. Local authorities should encourage active travel and protect and increase green spaces in urban areas to make the healthy option the easy option
10. Medical staff, including doctors, nurses and dietitians, should themselves be assessed on their metabolic health and encouraged and helped to improve it, not just to set an example to patients but to optimise their own health and performance.

*Source*: Malhotra A. The 21-day immunity plan. United Kingdom: Yellow Kite; 2021.

### The solutions

There was never any evidence justifying any COVID-19 vaccine mandates, passports or any of the other coercive measures adopted by various governments worldwide. Every patient who was offered any COVID-19 vaccine should have been made aware of what their risk from COVID-19 is according to age and risk factors. In keeping with ethical medical practice, doctors should have informed patients of their absolute risk reduction for infection from previous more lethal variant being approximately 0.84% or 1 in 119 (based on non-transparent data) and that this level of protection only lasts for a few months. They should also have provided more precise and robust data on what the actual absolute individual risk reduction of COVID-19 death from the vaccine is, what the true rates of serious adverse events (such as permanent disability, hospitalisation or death) are. It is only when doctors and patients have all this information that they can then be empowered to have frank decision making conversations on whether any treatment – including this vaccine – is right for them.

The profession must explain that optimising metabolic health will give patients the best chance for ensuring they are not just resilient to infection but reducing their risk of chronic disease including heart disease, cancer and dementia.

The time has come to stop misleading evidence flowing downstream into media reporting and clinical decision making and resulting in unethical and unscientific policy decisions. It’s time for real evidence-based medicine (Box 4^64^).

BOX 4 Defining real evidence-based medicine and actions to deliver it.

1. Is the application of individual clinical expertise with best available evidence and taking into consideration patient preferences and values in order to improve patient outcomes (relieve suffering and pain, treat illness and address risks to health)
2. Makes the ethical care of the patient it’s top priority
3. Demands individualised evidence in a format that clinicians and patients can understand
4. Is characterised by expert judgement rather than mechanical rule following
5. Shares decisions with patients through meaningful conversations
6. Builds on a strong clinician–patient relationship and the human aspect of care
7. Applies these principles at community level for evidence-based public health

**Actions to deliver real evidence-based medicine**

1. Although the pharmaceutical industry plays an important role in developing new drugs, they should play no role in testing them
2. All results of all trials that involve humans must be made publicly available
3. Regulators such as the FDA and MHRA must be publicly funded, and not receive any money from the pharmaceutical industry
4. Independent researchers must increasingly shape the production, synthesis and dissemination of high-quality clinical and public health evidence
5. Medical education should not be funded or sponsored by the pharmaceutical industry
6. Patients must demand better evidence, better presented (using absolute and not relative risk), better explained and applied in a more personalised way

*Source*: Adapted from Greenhalgh T, Howick J, Maskrey N. Evidence based medicine Renaissance Group. Evidence based medicine: A movement in crisis? *BMJ*. 2014;348:g3725. https://doi.org/10.1136/bmj.g3725

There is also a strong scientific, ethical and moral case to be made that the current mRNA vaccine administration must stop until Pfizer releases all the raw data for independent scrutiny.^30^ This will allow a more accurate understanding of which groups are more likely to potentially benefit from the vaccine versus those who are more likely to be harmed.

Given all the recent well-documented aforementioned shortcomings in medical research integrity (including that possibly half the published medical literature ‘may simply be untrue’), the editor of the *Lancet* Richard Horton wrote in 2015 that science has taken a turn towards darkness and asked who was going to take the first step in cleaning up the system.^65^ The unprecedented roll-out of an emergency use authorisation vaccine without access to the raw data, with increasing evidence of significant harms, compounded by mandates that appear to serve no purpose other than to bolster profits of the drug industry, have highlighted modern medicine’s worst failings on an epic scale, with additional catastrophic harms to trust in public health.

We must use this as an opportunity to transform the system to produce better doctors, better decision making, healthier patients and restore trust in medicine and public health. Until all the raw data on the mRNA COVID-19 vaccines have been independently analysed, any claims purporting that they confer a net benefit to humankind cannot be considered to be evidence-based.
